# Reducing HIV Vulnerability Through a Multilevel Life Skills Intervention for Adolescent Men (The iREACH Project): Protocol for a Randomized Controlled Trial

**DOI:** 10.2196/10174

**Published:** 2018-07-10

**Authors:** Jose Bauermeister, Patrick S Sullivan, Laura Gravens, James Wolfe, Kristina Countryman, Neena Smith-Bankhead, Ryan A Drab, Gregory Sallabank, Jordan D Helms, Kristie Khatibi, Rebecca Filipowicz, Keith Joseph Horvath, Erin Bonar, Amanda Castel, Lisa Hightow-Weidman, Jodie Guest, Rob Stephenson

**Affiliations:** ^1^ School of Nursing University of Pennsylvania Philadelphia, PA United States; ^2^ Rollins School of Public Health Emory University Atlanta, GA United States; ^3^ School of Nursing University of Michigan Ann Arbor, MI United States; ^4^ School of Public Health Division of Epidemiology and Community Health University of Minnesota Minneapolis, MN United States; ^5^ School of Medicine University of Michigan Ann Arbor, MI United States; ^6^ Milken Institute School of Public Health George Washington University Washington, DC United States; ^7^ School of Medicine University of North Carolina Chapel Hill, NC United States

**Keywords:** prevention, mHealth, adolescence, LGBTQ, sexuality, life skills, HIV

## Abstract

**Background:**

Few HIV interventions have demonstrated efficacy in reducing HIV risk among adolescent men who have sex with men (AMSM), and fewer still have recognized the unique needs of AMSM based on race/ethnicity or geographical setting. Recognizing that youths’ HIV vulnerability is intricately tied to their development and social context, delivering life skills training during adolescence might delay the onset or reduce the consequences of risk factors for HIV acquisition and equip AMSM with the skills to navigate HIV prevention. This protocol describes the development and testing of iREACH, an online multilevel life skills intervention for AMSM.

**Objective:**

This randomized controlled trial (RCT) aims to test the efficacy of an online-delivered life skills intervention, iREACH, on cognitive and behavioral HIV-related outcomes for AMSM.

**Methods:**

iREACH is a prospective RCT of approximately 600 cisgender adolescent males aged 13 to 18 years who report same-sex attractions. The intervention will be tested with a racial/ethnically diverse sample (≥50% racial/ethnic minority) of AMSM living in four regions in the United States: (1) Chicago to Detroit, (2) Washington, DC to Atlanta, (3) San Francisco to San Diego, and (4) Memphis to New Orleans.

**Results:**

This project is currently recruiting participants. Recruitment began in March 2018.

**Conclusions:**

iREACH represents a significant innovation in the development and testing of a tailored life skills-focused intervention for AMSM, and has the potential to fill a significant gap in HIV prevention intervention programming and research for AMSM.

**Registered Report Identifier:**

RR1-10.2196/10174

## Introduction

### Background

Between 2000 and 2010, the annual number of new HIV diagnoses among young men who have sex with men (YMSM; age 13-24 years) in the United States more than doubled [1]. In 2016, youth aged 13 to 24 years made up 21% of all new HIV diagnoses in the United States. Most (81%) of those new diagnoses occurred among young gay and bisexual men. Young black/African American and Hispanic/Latino gay and bisexual men were especially affected [1]. The HIV epidemic among YMSM is characterized by strong racial and ethnic disparities in HIV incidence. Data from the National HIV Behavioral Survey showed 26% of African American youth surveyed (aged 18-24 years) tested HIV positive compared to only 3% of white youth aged 18 to 24 years [2]. Although initially attributed to greater engagement in risk behaviors among racial/ethnic minorities, recent analyses [3-7] illustrate that racial/ethnic disparities in HIV are likely driven by social determinants of health, including access to health insurance and social network properties, and by more limited coverage of effective HIV treatment for those living with HIV. These findings underscore the importance of understanding and addressing the structural factors driving the HIV disparities faced by marginalized YMSM in the United States.

Few HIV interventions have demonstrated efficacy for reducing HIV risk among adolescent men who have sex with men (AMSM). We employ the term AMSM to refer to cisgender males aged 13 to 18 years who may express same-sex attractions and/or engage in same-sex behaviors, yet may or may not identify as gay, bisexual, queer, and/or questioning. AMSM also represent a younger age range than YMSM, which typically includes ages up to 24 years. Mustanski [8] notes that relative to HIV prevention research with adult populations, AMSM have received less research attention, with a paucity of longitudinal studies with follow-up periods of greater than 12 months or the testing of interventions that recognize the unique developmental context of AMSM. Recognizing that AMSM’s HIV vulnerability is intricately tied to their developmental stage and social context [9,10], research has posited that delivering life skills training during adolescence may delay the onset of or reduce the consequences of risk. The World Health Organization describes life skills as “the ability for adaptive and positive behavior that enables individuals to deal effectively with the demands and challenges of everyday life.” For AMSM, life skills training may include a set of resources tailored to their individual and social contexts, allowing them to learn about and manage their HIV risk. A review [11] concluded that behavioral interventions that teach life skills are highly effective for HIV risk reduction among adult men who have sex with men (MSM); however, there is limited evidence examining whether life skills training for AMSM is an efficacious HIV prevention strategy.

Life skills training programs may be suited to electronic delivery given the proven appeal of e-interventions among youth, the suitability for delivering tailored content specific to each user’s HIV risk behaviors and context, and the opportunity to reach AMSM residing in diverse geographic locations [3,4]. Furthermore, given that MSM often rely on online technologies to build their social and sexual networks, receive social support, and obtain relevant health information [12-14], an e-delivered platform may reach AMSM who might otherwise not be able to access LGBTQ+–friendly (LGBTQ+: lesbian, gay, bisexual, transgender, queer, and additional identities) resources and services (eg, rural AMSM). In this protocol paper, we describe our plans to test the efficacy of iREACH, a life skills training Web-based app designed for racially and ethnically diverse AMSM living in four diverse regions of the United States that include rural and urban settings.

### Objectives

The primary objective of this randomized controlled trial (RCT) is to test the efficacy of an e-delivered life skills intervention, iREACH, on cognitive and behavioral HIV-related outcomes for AMSM. We will recruit a large and diverse sample of AMSM (N=600; ≤50% non-Hispanic white) living in four regions disproportionately burdened by HIV prevalence across the United States. We have two secondary aims for this project: (1) to examine the differential efficacy of iREACH in shaping the psychosocial mediators (eg, personal competency) associated with our outcomes based on engagement with the intervention, and (2) to explore how socioecological determinants at the individual (eg, race/ethnicity, urbanity) and regional (eg, socioeconomic disadvantage, HIV prevalence) level are associated with intervention efficacy.

## Methods

### Trial Design

We will conduct a prospective RCT of 600 online-recruited cisgender AMSM (age 13-18 years) followed for 12 months with study assessments at each 3-month interval. A racially/ethnically diverse sample (at least 50% racial/ethnic minority) of AMSM living in four regions in the United States: (1) Chicago, IL to Detroit, MI; (2) Washington, DC to Atlanta, GA; (3) San Francisco, CA to San Diego, CA; and (4) Memphis, TN to New Orleans, LA. Regions were identified by inspection of HIV prevalence rate maps on AIDSVu.org. Eligible counties are those that include the major interstate highway that connects the two anchor cities (ie, I-94 for Chicago to Detroit; I-95 for Washington, DC to Atlanta; I-5 for San Francisco to San Diego; and I-55 from Memphis to New Orleans). Each region includes urban, suburban, and rural counties, as classified by the 2006 National Center for Health Statistics urban-rural classification scheme for counties [15].

### Eligibility Criteria

Eligible individuals must (1) have been assigned a male sex at birth and identify as male at the time of enrollment into the study (cisgender male), (2) be between the ages of 13 and 18 years (inclusive), (3) speak and read English, (4) report same-sex attractions and/or behaviors, (5) have access to the Internet, (6) live in one of the zip codes at least partially contained in the 109 counties included in the four regions selected for this trial, and (7) self-report as HIV-negative at time of enrollment.

### Recruitment, Screening, Consent, and Enrollment

**Figure 1:**
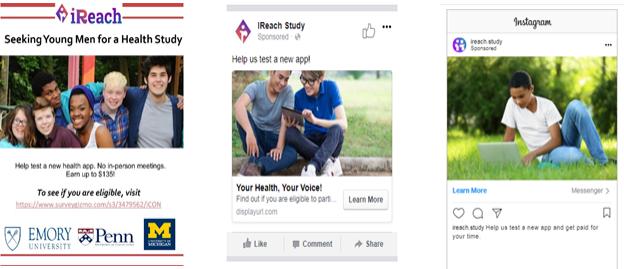
Examples of advertisements used to recruit racial/ethnically diverse adolescent men who have sex with men.

Potential participants will click on targeted banner advertisements (Figure 1) based on our eligibility criteria (eg, sex, age, region) placed on commonly used social media sites (ie, Facebook), which will direct them to a home page containing basic study information. We will also recruit through community events (eg, LGBTQ+ pride events). Interested individuals will consent to complete an online screener. As part of the screener, we will verify that the participant lives in one of the counties selected for this trial based on their reported zip code. Individuals who do not meet the eligibility criteria will see a screen that thanks them for their interest and provides HIV testing and counseling information and resources. We will not indicate why they were ineligible to avoid unintentional disclosure of their study involvement and to protect against fraud*.* Eligible individuals will be taken to the study consent form (a waiver of parental consent has been obtained for minor participants). AMSM who do not consent will be taken to a screen thanking them for their interest. AMSM who consent will submit a cell phone number and their cell phone carrier as part of the registration process, an email and/or short message service (SMS) text message containing a code will be immediately sent to verify the user. This process has been found to be acceptable among adolescents [16] and MSM [17,18] in previous studies. After verification, participants will be asked to provide their contact information, including an email address, a cell phone number, social media handles, and a mailing address, and will be asked to provide a nickname or name of choice to be referred to throughout the study. Participants will then be directed to the baseline survey, which is estimated to take approximately 30 minutes to complete.

Completed baseline survey data and participant information will be manually reviewed and checked for duplications or possible fraud by study staff members. Responses from the screening survey will be checked against responses in the baseline survey (eg, age) to ensure consistency, and IP addresses, email addresses, and phone numbers will be reviewed to check for multiple registrations. Proxy IP addresses will be flagged for further scrutiny, and reviews of the baseline survey data will be conducted to check for suspicious response patterns and realistic completion times. Within 48 hours, participants will receive their log-in credentials, their incentive payment for completion of the baseline survey, and will be assigned in a 1:1 ratio for the intervention or attention control condition using stratified randomization [19] by race and region. Screener and baseline data will be used by iREACH to inform personalized, tailored content for AMSM assigned to the intervention condition.

### Intervention Content: iREACH

**Figure 2:**
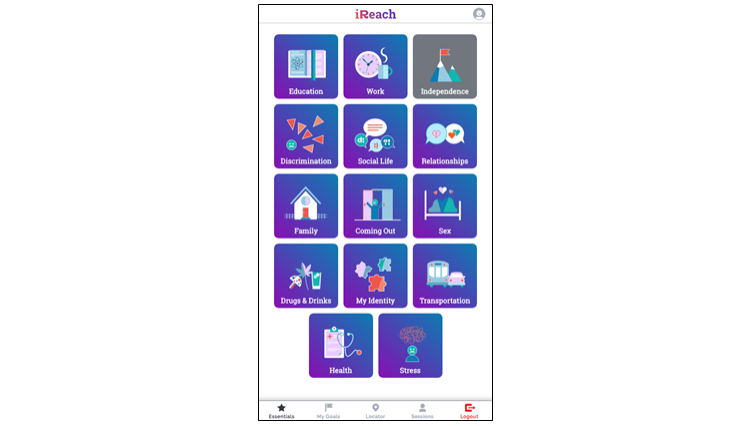
Screen image of iREACH intervention app.

iREACH (Figure 2) is a tailored Web app intervention for AMSM aged 13 to 18 years. The intervention component of the app aims to facilitate participants lowering their vulnerability to HIV infection by (1) providing life skills educational modules tailored to their unique needs and characteristics, (2) setting goals and encouraging participants to use relevant services available locally to help achieve them, and (3) accessing LGBTQ+–welcoming resources across the life skills areas. Individuals in the experimental arm will have access to iREACH over the 12 months of the study. Within the Web app, they will learn life skills content through activity-based learning across 14 key life areas, set goals in those areas and monitor progress toward these goals, work on these goals using the peer mentor video chat feature, and locate nearby LGBTQ+–welcoming community resources to achieve these goals. We describe iREACH’s main components subsequently.

#### Essentials

**Figure 3:**
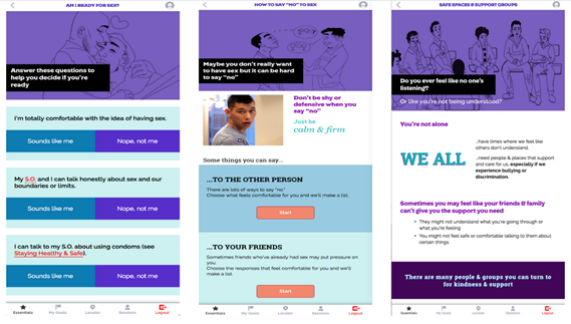
Screen images of iREACH intervention app illustrating age-appropriate literacy and interactive format. SO: significant other.

The life skills educational content in iREACH covers 14 topics. As shown in Figure 3, content is written at the eighth grade literacy level and presented in an interactive format, which includes infographics, GIF (Graphics Interchange Format) images, interactive activities, and accordion (drop-down) headers to improve the ease of navigation and level of cognitive effort. Tailored (ie, personalized) content is derived from AMSM’s baseline sociodemographic characteristics (eg, age, region) and answers to follow-up surveys inform new personalized content. For example, although we will only enroll participants who identify as male at baseline, iREACH is responsive to the gender development of AMSM during the study: if a participant identifies as transgender or gender nonconforming during the intervention, they can edit their profile name and preferred pronouns. Similarly, if a participant reports being newly diagnosed with HIV during the study, iREACH is designed to provide HIV-specific content (eg, how to keep up with their treatment plan, how Ryan White funds can assist with medication, transportation, and social support).

#### Goal Setting

**Figure 4:**
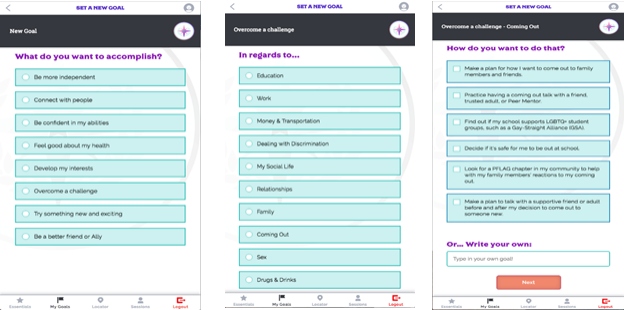
Screen images of iREACH intervention app illustrating goal-setting activities.

**Figure 5:**
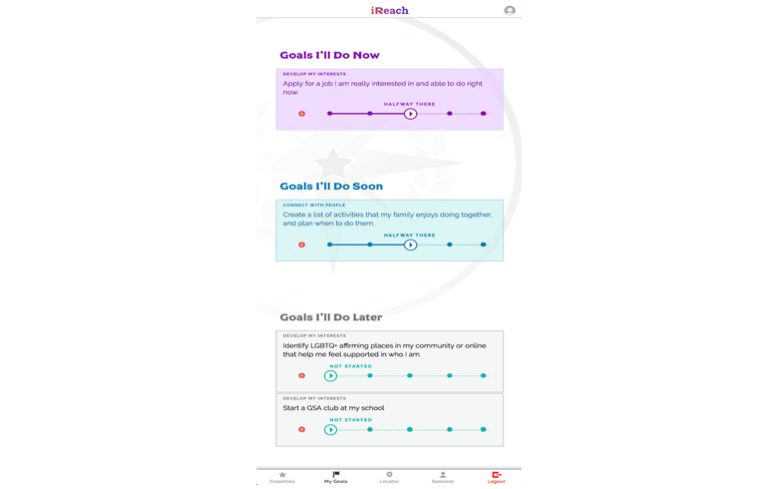
Screen image of iREACH intervention app illustrating goal progress tracking.

Participants will be able to set, monitor, and track their goals through the “My Goals” component of the Web app. The variety of possible goals is based on AMSM’s wide range of potential needs and experiences across the four regions. As shown in Figure 4, participants select the primary goal (eg, autonomy, competence, relatedness, and self-actualization) they wish to work on, followed by the relevant life skills domain (eg, work, relationships, and sex). Participants can then select from a list of prepopulated common goals or can create their own goal, and determine whether it is a short-term (now), medium-term (soon), or long-term (later) goal. Using a progress navigation bar, participants can track their progress on their goals (Figure 5), delete goals that are no longer relevant, and review goals marked as completed. Participants may also review and receive feedback on their goals through peer mentor sessions (described subsequently).

#### Local Resources

Participants who click on the “Locator” button will access a prepopulated list of national (eg, crisis hotlines) and local resources (eg, gay-straight alliances, HIV testing locations). We identified 1833 eligible resources across our four study regions; each resource was verified through mailers and phone calls using protocols adapted from HIV testing [20] and pre-exposure prophylaxis (PrEP) [21] locators. A random subsample (20%) of resources will be selected for review every 6 months and updated as necessary.

The Locator section filters resources for the participants' regions automatically and clusters the resources linked to each life skill area. Participants can further refine the resources based on their county, zip code, and/or current distance to the resource (as computed by their browser’s geolocation if activated). If a local resource does not exist in their area or if a participants moves out of the designed regions during the study, the Locator will provide national hotlines and websites. We detail the proportion of services identified, verified and included in our Locator in Table 1.

#### Peer Mentors

Building on the Web app’s life skills content and goal-setting structure, iREACH acknowledges that AMSM’s achievement of these goals can be enhanced by role playing and coaching. LGBTQ+ supportive peers may be limited in some communities; therefore, iREACH provides participants with access to trained peer mentors who can help AMSM personalize the life skills lessons, set new goals and/or support those already identified, and provide peer-to-peer social support. Participants can schedule and attend these peer mentor appointments (approximately 30 minutes, on average) through the “Sessions” button.

These sessions are housed within the VSee video chat telemedicine platform, accessible from their Web app. AMSM concerned about privacy may use VSee in four ways: (1) video in which both they and the peer mentor can be seen, (2) video in which only the peer mentor can be seen, (3) audio only, or (4) a text-only chat interface. Peer mentors (YMSM aged 18-29 years) will be trained to use motivational interviewing principles in their exchanges with AMSM, and supervised by study team members experienced in motivational interviewing and counseling (eg, psychologist, professional counselor). Peer mentors will use a social problem-solving approach to help participants set goals that are achievable and moderately challenging and to identify potential solutions to problems. Participants will be able to schedule a maximum of three sessions per week. This service will be available throughout the full year of their assignment to the intervention.

**Table 1:** Number of local resources identified and included in the iREACH locator by region^a^.

Region	HIV services, n (%)	Mental health and substance use, n (%)	LGBTQ+^b^ resources, n (%)	Food pantry, n (%)	Shelters and housing, n (%)	Intimate partner violence resource, n (%)	Total resources in region (N=1933), n (%)
Chicago, IL to Detroit, MI	376 (47.1)	472 (59.1)	152 (19.0)	31 (3.9)	91 (11.4)	103 (12.9)	798 (43.5)
Washington DC to Atlanta, GA	233 (43.7)	317 (59.5)	123 (23.1)	16 (3.0)	31 (5.8)	37 (6.9)	533 (29.1)
San Francisco, CA to San Diego, CA	154 (47.0)	239 (72.9)	81 (24.7)	11 (3.4)	28 (8.5)	32 (9.8)	328 (17.9)
Memphis, TN to New Orleans, LA	86 (50.6)	82 (48.2)	7 (4.1)	6 (3.5)	8 (4.7)	9 (5.3)	170 (9.3)
National hotlines	0 (0.0)	3 (75.1)	3 (75.4)	2 (50.4)	1 (25.2)	2 (50.3)	4 (.22)

^a^Resources often offer multiple types of services; percentages reflect the proportion of local resources offering a specific type of resource. Regions comprise all counties touching the major interstate corridor connecting the two anchor cities.

^b^LGBTQ+: lesbian, gay, bisexual, transgender, queer, and additional identities.

#### Additional Engagement

**Figure 6:**
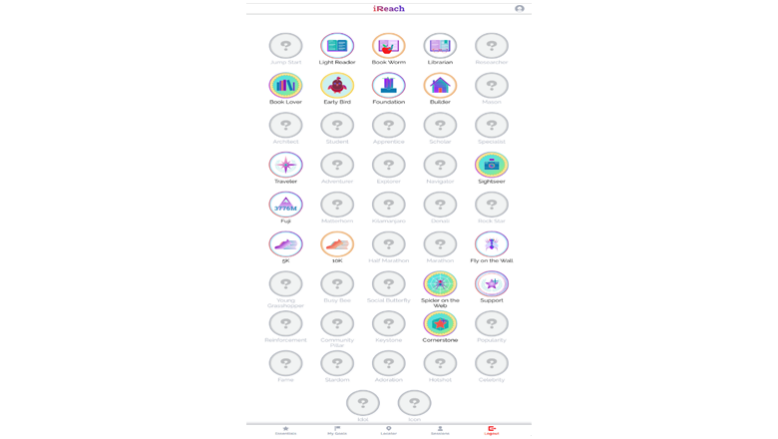
Screen image of iREACH intervention app illustrating badge earning.

To promote on-going user engagement, intervention participants will also gain badges (Figure 6) to reinforce continued participation on the site [22,23]. Badges are unlocked as participants complete tasks on the Web app (eg, setting goals, reading content, scheduling peer mentor sessions), and continuously engage in the site (eg, returning to the Web app, using it on weekdays and/or weekends, logging on the site on different hours of the day). Participants also can access a message board where they can start and discuss topics with each other. Peer mentors will monitor these boards and facilitate discussions as needed.

### Information-Only Attention Control Arm

**Figure 7:**
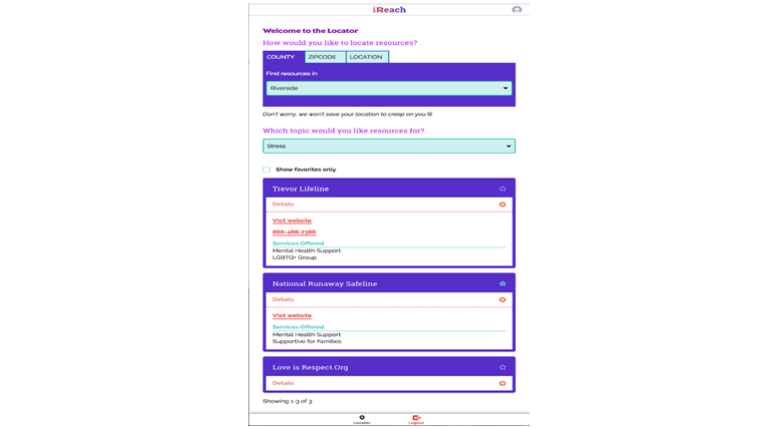
Screen images of information-only attention control condition illustrating service locator information.

Those AMSM assigned to the attention control arm (N=300) will only receive access to the “Locator” component of the intervention (Figure 7). Although the provision of a service locator is a form of an intervention, albeit weak, and it may decrease our ability to detect intervention effect, we felt that withholding referrals to services would be unethical given AMSM’s vulnerability to HIV and sexually transmitted infections (STIs). At the end of the RCT, participants in the attention control condition will be given full access to the iREACH intervention for 3 months.

### Outcomes

Because our participants will be ages 13 to 18 years and many might not have begun sexual activity or used HIV prevention services, we set our primary outcomes as cognitive factors linked to the ability to use HIV prevention and behavioral intentions to use HIV prevention. Our secondary outcomes are behavioral factors. Cognitive factors can be measured for all AMSM, regardless of whether they have initiated sexual activity with partners, and are broadly grouped into (1) knowledge, (2) attitudes, (3) norms, (4) self-efficacy, and (5) perceived behavioral capacity. Behavioral intention outcomes include self-reported intentions to adopt HIV risk reduction strategies and HIV prevention services. We also include secondary outcomes that apply to the subset of AMSM who are sexually active, and include self-reported HIV risk-taking behavior (both sexual and drug/alcohol use) and the use of HIV prevention services (eg, testing) and prevention activities (eg, abstinence, condoms, PrEP). We detail study measures in Table 2.

**Table 2:** Measures planned for a randomized controlled trial of a life skills intervention for adolescent men who have sex with men in the United States.

Domain		Assessment time					
		Baseline	Month 3^a^	Month 6	Month 9	Month 12	Month 15^b^
**Primary outcomes**							
	HIV knowledge [24]	X		X		X	X
	Condom use/communication efficacy [16]	X		X		X	X
	HIV/STI testing	X		X		X	X
**Outcomes required for analysis**							
	Demographics	X		X		X	X
	Patient provider communication around sexual orientation [25]		X		X		
	Stigma [26]		X		X		
	Resilience [27]		X		X		
	Psychological needs [28]	X		X		X	X
	Future life goals [29]		X		X		
**Secondary outcomes**							
	Internalized homonegativity [30]	X		X		X	X
	PrEP use and willingness [31]	X		X		X	X
	Sex behaviors [32,33]	X		X		X	X
	Substance abuse [34,35]	X		X		X	X
	Depression [36]	X		X		X	X
	Anxiety [37]	X		X		X	X
	Self-Esteem [38]	X		X		X	X
**Covariates**							
	Peer influence [33]		X		X		
	Family support [39]		X		X		
	Discrimination [40]		X		X		
	Online behaviors [41,42]		X		X		
	Societal reaction to sexual orientation [43]		X		X		
	Ethnicity beliefs [44]		X		X		
	Relationship history [45]	X		X		X	X
	Intervention acceptability [46,47]		X				X

^a^The iREACH intervention question set will only be available to the intervention group for month 3.

^b^Only participants initially assigned to the control condition will complete survey at month 15 after receiving access to the iREACH intervention.

### Incentives

Participants receive US $30 for the baseline and 12-month follow-up and US $25 for the 3-, 6-, and 9-month follow-ups. Control participants will receive US $30 for completing the 15-month follow-up. These incentives are small enough to avoid coercion, yet sufficiently substantial to promote retention.

### Recruitment and Randomization

#### Sample Composition

We used Census information on population structure by race/ethnicity for each region to inform our recruitment goals. Briefly, using 2010 Census data, we calculated the number of men in the eligible age group and by race/ethnicity in each region (Table 3). We then used data from the Youth Behavioral Risk Factor Surveillance System [48] to estimate the proportion of AMSM in each age group who could meet eligibility criteria based on same-sex behavior, identity, or attraction for our study, and applied that age-specific proportion to the estimated total AMSM population by race in each region. This resulted in an estimated number of eligible AMSM per region and racial/ethnic group. Within each region, we calculated a proportional recruitment target under the assumption that the 150 AMSM in that region would be recruited proportional to their population prevalence.

**Table 3:** Estimated eligible adolescent men who have sex with men by race and ethnicity and proportional and planned study enrollment for each region.

Race/ethnicity	Region^a^, n														
	San Francisco/San Diego			Atlanta/Washington, DC			Detroit/Chicago			Memphis/New Orleans				Total^b^	
	Est^c^	Prop^d^	Goal^e^	Est	Prop	Goal	Est	Prop	Goal		Est	Prop	Goal		
Hispanic	41,515	49	29	5732	16	29	6186	23	29		742	8	28		115
White	59,424	70	32	30,531	84	31	21,777	82	31		6974	75	31		125
Black/African American	6434	8	42	13,488	37	38	9053	34	37		5805	62	38		155
Asian	14,185	17	12	2988	8	12	1822	7	13		268	3	13		50
American Indian or Alaska Native	664	1	11	168	1	13	112	1	13		28	0	13		50
Native Hawaiian/Other Pacific Islander	362	0	11	24	0	13	0	0	13		1	0	13		50
Multiracial	4349	5	13	1355	4	14	888	3	14		202	2	14		55
Total	126,933	150	150	54,286	150	150	39,838	150	150		14,020	150	150		600

^a^Regions comprise all counties touching the major interstate corridor connecting the two anchor cities.

^b^Totals within race/ethnicity groups may not sum to total planned enrollment due to rounding.

^c^Est: estimate. Estimated total adolescent MSM (aged 13-18 years) in the region who have same-sex sexual experience, same-sex attraction, or are gay/bisexually identified.

^d^Prop: proportion. Reflects the target recruitment for each racial/ethnic subgroup, assuming that enrollment is evenly distributed by region, and proportionally distributed by population prevalence of eligible AMSM within region.

^e^Goal: trial recruitment goal.

To recruit a sample that is diverse in terms of race/ethnicity, we will need to substantially oversample some racial/ethnic subgroups based on these calculations. To achieve a sample that is 50% or more nonwhite adolescent men, we will oversample Hispanic AMSM by a factor of 1.2, Asian MSM by a factor of 1.5, multiracial AMSM by a factor of 3.8, Native American AMSM by a factor of 25.6, and Native Hawaiian/Pacific Islander MSM by a factor of 100 (Table 4). Therefore, we have budgeted more resources for recruitment in these subgroups, and will direct substantial recruitment focus toward these groups through selection of race/ethnicity concordant models in advertisements and through identifying community events or support groups that are especially relevant to these subgroups [49].

**Table 4:** Overall recruitment goals of adolescent men who have sex with men (AMSM) based on allocation based on population prevalence, planned recruitment, and ratio of planned to proportional allocations for a randomized controlled trial of a life skills intervention.

Race/ethnicity	Proportional^a^, n	Planned^b^, n	Relative recruitment^c^, n
Hispanic	96	115	1.2
White	311	125	0.4
African American/black	141	155	1.1
Asian	35	50	1.5
American Indian or Alaska Native	2	50	25.6^d^
Native Hawaiian/Other Pacific Islander	1	50	>100.0^d^
Multiracial	14	55	3.8^d^
Total	—	600	—

^a^Proportional recruitment is determined by multiplying the estimated proportion of all AMSM in the four regions by the total study enrollment (N=600).

^b^Planned enrollment is an empirically determined set of requirement targets.

^c^Relative recruitment is the ratio of the planned/proportional recruitment numbers, representing the relative under- or overrepresentation of the population in the planned sample, relative to their representation in the overall population of the study areas.

^d^These relative recruitment values are calculated from proportional recruitment numbers with more precision than those displayed in the proportional column (eg, for Native Hawaiian/Other Pacific Islander, the proportional sample is calculated as 0.0016 person, but it is depicted as 1 since fractional recruitment of participants is not possible) . Therefore, the relative recruitment differs from the simple ratio of the planned to proportional for these groups.

These data also make clear that the distribution of participants will not be equal across the four geographic regions within racial/ethnic groups; therefore, we will stratify randomization within race and region. Based on estimated AMSM population sizes and historical data on sampling fractions, we anticipate more than 35% of participants will come from rural areas [50]. We recognize the challenge of recruiting early adolescents given that same-sex attraction and same-sex sexual experience become more common with age. Therefore, we realistically expect to include a greater number of participants in the older age range.

#### Recruitment

Recruitment will utilize both virtual and physical venues. Targeted advertisements, representing age and ethnic diversity, will be promoted on Facebook, Instagram, Snapchat, and organizational websites. In addition, supplemental advertising on other social media platforms (YouTube, Tumblr, Twitter, and Reddit) may be used to expand recruitment efforts. The social media campaigns will be monitored and adjusted throughout the recruitment phase. Across all regions, physical recruitment materials will be developed and distributed among organizations that serve and support LGBTQ+ youth (eg, homeless youth organizations, community LGBTQ+ centers, HIV resources). Physical recruitment materials will also be distributed during large community events, including pride festivals in the study regions.

#### Strategies to Ensure Sample Diversity

We will develop ads that promote AMSM’s interest by including diverse images of youth (Figure 1) and targeting specific sociodemographic characteristics and interests.

During the formative phase of the study, facilitated focus group discussions were conducted online to identify the most effective recruitment strategies to optimize diversity and to ensure that culturally appropriate strategies are employed to engage and retain enough Asian/Pacific Islander, Native American, and Alaskan Native participants. These include promoting the study to agencies, social media groups, and in social media forums highly utilized by youth in these specific ethnic groups, and incorporating recruitment messages that encourage community engagement. Materials will avoid identifying candidates as AMSM in the recruitment text to avoid unintended disclosure.

#### Retention

To be evaluated as potential “best-evidence” interventions through the Centers for Disease Controls and Prevention’s Prevention Synthesis Research activity [51], data must be available for at least a single follow-up time point for more than 70% of participants. As indicated subsequently, a detailed retention plan for the study will draw on previously successful retention protocols to achieve 80% or more retention at the first follow-up visit. We will use successful best practices from previous studies [17,49] to retain participants (eg, comprehensive locator information that includes participants’ cell phone number, email, Facebook and/or other social media usernames), while being sensitive to undue disclosure of AMSM participating in the study. In addition, we allow participants to specify the day of the week and time of day when they would like to receive electronic follow-up surveys [17].

We have a preplanned schedule of follow-up assessments utilizing a variety of methods. Initially, a respondent who does not respond to an electronic notification that a survey is due will automatically receive additional notifications 48 hours after the initial notification the survey is available. If the participant has still not completed the assessment 7 days after the third electronic notification, the retention activities are escalated to a research staff member who will use the participant’s contact preferences provided on registration (eg, by SMS text message). If still unresponsive, other available contact information (eg, phone call) will be used. Each contact is logged in an electronic retention system (Study Management and Retention Toolkit [SMART]) developed by Emory University [17,18,52]. The SMART system also maintains electronic lists of participants’ retention status, and automatically creates notification lists for retention staff to ensure that a systematic process is followed and carefully documented for retention.

### Statistical Methods

The primary outcome analyses seek to test the efficacy of iREACH compared to the information-only control condition to improve cognitive factors and behavioral intentions (eg, comfort discussing sexuality, HIV prevention attitudes, norms, self-efficacy) and behavioral factors (eg, condom use, HIV testing, PrEP use). Psychosocial and demographic characteristics will be described for all participants and by intervention group. These will be compared between treatment groups using *t* tests or Wilcoxon rank sum tests for continuous variables and chi-square tests for categorical variables. We will determine from the analyses stratified by treatment arm whether or not a failure of randomization occurred. We will use the general framework of generalized linear mixed models to test for intervention effects over time. For some binary outcomes, such as HIV testing, we will perform an aggregate analysis after collapsing across the repeated measures using simple logistic regression comparing whether the probability of having tested at least once over the entire follow-up period is different across treatment groups. To ensure robustness, we will also apply an exchangeable working correlation structure to its corresponding generalized estimating equation model.

#### Sample Size and Power Calculations for Primary Analyses

Our expected sample size for analyses across both conditions is N=600 (intervention: n=300; control: n=300), assuming a 15% to 20% loss to follow-up. We estimated the minimum detectable effect sizes at 80% power, for comparisons of the two groups for the primary cognitive and behavioral intentions outcomes. For mean differences, our sample size calculations are based on a two-sample *t* test assuming equal variance using a two-sided significance of .05. At 80% power, we can detect a between-arm difference of *d*=0.22 at the final follow-up. For repeated measure analyses, assuming a within-person correlation of .25, we would be able to detect a difference of 0.08. For proportions, our sample size calculations are based on a two-sample test of proportions using a two-sided significance of .05. To have 80% power to compare the intervention to the control group, we require at least 500 participants to find a 12.5% difference between arms in cross-sectional analyses. Assuming within-person correlation of .25, we can detect an 8.8% difference.

#### Secondary Analysis

To examine the effects of our intervention on the psychosocial correlates (eg, personal competence), we will run a regression with only group assignment in the model. Among participants assigned to the intervention arm, we will test whether the intervention effects vary as a function of AMSM’s varying engagement with the intervention as measured by paradata metrics (eg, frequency of site log-ins, time spent on intervention components). Among participants assigned to the control arm, we will describe AMSM’s varying engagement with the resource guide as measured by paradata metrics (eg, frequency of site log-ins) and test whether engagement is associated with changes over time within the control arm. Finally, we will use multilevel models to examine how regional characteristics influence AMSM’s outcomes [53]. We will link individual and regional level data using participants’ residential address at enrollment. Our regional unit of analysis will be county-level to ensure enough participants per region and to avoid inadvertent identification of participants. Exploratory analyses will examine whether county-level characteristics (eg, economic disadvantage, racial composition, HIV prevalence) are associated with individual-level outcomes. Analyses will be adapted for binary, count, or continuous outcomes accordingly.

#### Intervention Exposure, Fidelity, and Dosage

We will measure intervention exposure using paradata from the intervention, including counts of user sessions, session lengths, pages visited, and functions utilized [54]. For the peer mentor component, we will record the number and duration of peer mentor sessions, as well as the domains covered in these sessions. This information will assist in examining whether intervention dosage influences the efficacy of the intervention, and inform wider implementation and scalability.

## Results

The protocol has been reviewed and approved by the University of Pennsylvania Institutional Review Board (825686) and is registered on ClinicalTrials.gov (NCT03155841).

This project is currently recruiting participants. Recruitment began in March 2018.

## Discussion

### Limitations and Anticipated Challenges

There are several potential challenges to the success of our trial. First, we propose to recruit a diverse (in terms of race/ethnicity, rurality, and socioeconomic status) sample of adolescents aged 13 to 18 years. This poses two potential challenges. First, we may experience more success in recruiting AMSM at the older ages of this range (eg, 16 years and older). To counteract this challenge, we have elicited feedback from AMSM during our formative planning and have planned for a broad range of social media outlets utilized commonly by these age groups in our recruitment. We will also leverage youth-focused social media outlets in each of the four sampled regions when available. Second, we recognize that AMSM living in the urban centers of each of our sampled regions and/or who are non-Hispanic white might be easier to recruit into the study. There is now strong evidence of high levels of Internet, personal computer, and mobile phone use across all race and ethnic groups [41], indicating that our modes of recruitment should not bias toward any particular demographic. We will ensure racial and ethnic representation in all advertising. Many social media outlets allow advertising targeted by zip codes, allowing us to target recruitment ads to all zip codes in each of our sampled regions. We will monitor the characteristics of our enrolled sample closely; if we are enrolling urban youth at a faster pace than rural youth then we will increase our advertising efforts in rural zip codes. Third, given time and resource constraints, our intervention focuses on English-speaking AMSM. Although this decision may exclude Spanish-only speakers, recent Census data suggests that 88% of Hispanic youth younger than 17 years in the United States are proficient in English [55]. If the intervention is found to be efficacious, we will explore opportunities to translate the intervention. Fourth, there is a possibility that peer mentor sessions may be popular among AMSM and might require us to increase the number of available slots per day. If so, we will adjust our schedule and hire/train new peer mentors to meet the demand. Finally, we are vigilant of events that may lead to unintended disclosure of sexuality, behavior, and research participation to the participant’s parents/guardians. To minimize the possibility of unintended disclosures, we have integrated widely used Web privacy features, such as password authentication, automatically logging users out after a period of inactivity, and not including sensitive details in communications sent to participants. We have also structured the intervention to give participants some control over their own privacy, such as allowing them to indicate preferred methods of receiving communication (ie SMS text message, email, or social media private message), and remind participants to be active in protecting their information. If a parent or guardian becomes aware of AMSM’s participation in the trial and chooses to reach out to the study team, a member of the research team will be available to speak with the parent/guardian; however, to protect AMSM’s privacy, we will not confirm their child’s participation in the trial or share data with parents.

### Conclusions

As the numbers of HIV-infected AMSM continue to grow, innovative methods to scale up HIV prevention to AMSM are required. A life skills intervention delivered to a diverse sample of AMSM may reduce the existing HIV disparities across social categories (eg, race/ethnicity, rurality, socioeconomic status) by promoting equitable access to psychosocial and sexual health resources, and by reducing HIV-related risk factors that may be learned in adolescence and sustained across adulthood. The tools and framework used in this project may be directly applicable to US-based studies of this population. Along with promoting HIV risk reduction, our most significant contribution will be the development of an online intervention that builds on AMSM’s life skills and addresses the psychosocial needs during this developmental period. In fact, given the dearth of studies focused on AMSM, our trial may represent one of the first systematic evaluations of a psychosocial and behavioral intervention for this age group.

## Abbreviations

AMSM: adolescent men who have sex with men
LGBTQ+: lesbian, gay, bisexual, transgender, queer, and additional identities
MSM: men who have sex with men
RCT: randomized controlled trial
SMART: Study Management and Retention Toolkit
SMS: short message service
STI: sexually transmitted infections
YMSM: young men who have sex with men
